# Vascular Stromal Fraction Augmented With Autologous Cartilage Particulate and Hyaluronic Acid Mesh for the Treatment of Focal Cartilage Lesions: Description of a Surgical Technique

**DOI:** 10.1016/j.eats.2025.103692

**Published:** 2025-07-12

**Authors:** Gustavo Álvarez, Viviana Cortes, Francisco Sánchez, Sebastían López, Agustín Zapata, Camilo Cabezas, Anwar Arabia

**Affiliations:** a Clínica de Artrosis, Medellín, Colombia; b Research Group in Articular Biotechnology and Advanced Cellular Therapies, BIOJOINTECH, Medellín, Colombia; c Universidad de Antioquia, Medellín, Colombia; d Clínica las Vegas, Medellín, Colombia

## Abstract

This study presents a surgical approach combining adipose-derived stromal vascular fraction, autologous cartilage particulate, and a hyaluronic acid mesh to treat focal cartilage lesions. Early findings indicate the method is safe and feasible, showing promise for enhancing cartilage regeneration and potentially delaying osteoarthritis progression. This integrated technique may offer improved clinical outcomes and quality of life for patients, although further research is necessary to validate long-term efficacy and refine surgical protocols.

Focal articular cartilage lesions are a common cause of pain and dysfunction in the active population, particularly athletes and young adults. These lesions represent a significant therapeutic challenge in orthopaedics because of the limited regenerative capacity of cartilage.

Various treatments for these types of lesions have been historically explored, including interventions such as chondroplasty, microfracture, and cartilage transplant. However, they are often insufficient to reverse cartilage damage, highlighting the need for effective and long-lasting biological strategies.

In recent years, biological therapies have emerged as a promising alternative for the treatment of cartilage lesions; adipose-derived stromal vascular fraction (SVF) has gained considerable attention because of its ability to provide an environment rich in mesenchymal stem cells^1^ and other cellular components that promote tissue repair and modulate inflammation.^2^ SVF can modulate inflammation and initiate regeneration in articular tissues through a paracrine effect. The chemokines released by SVF can slow degeneration and stimulate regeneration in joints.^3^

Studies have also shown recently that SVF, either injected intra-articularly or combined with other biological materials such as scaffolds designed to direct and position cells in specific areas, is effective in cartilage repair and can improve joint function and relieve pain in patients with knee osteoarthritis.^4^ However, evidence on the combination of SVF and autologous cartilage particulate for focal cartilage regeneration is still limited. This approach not only provides structural support through the particulate cartilage but also introduces a biologically active microenvironment that could overcome the limitations of traditional techniques by promoting more effective hyaline cartilage regeneration. Additionally, the combination with hyaluronic acid scaffolds has shown promising results in guiding cellular regeneration in specific areas, thereby enhancing the effectiveness of therapies.

## Surgical Technique

Indications for the described procedure are as follows: symptomatic patients, ideally young or middle aged (generally <55 years), with Outerbridge grade IV focal cartilage lesions measuring between 1 and 4 cm^2^; focal cartilage lesions that are contained or delimited by healthy cartilage; lesions that have failed to respond adequately to conservative treatments; and patients for whom previous treatments such as microfracture or osteochondral graft have failed. Contraindications consist of diffuse or uncontained cartilage lesions; uncorrected malalignment, joint instability, or generalized joint involvement; and patients with systemic diseases that compromise healing mechanisms, such as uncontrolled diabetes, active autoimmune diseases, or vascular disorders. However, the decision should be based on an individualized evaluation of each case.

### SVF Processing

Our technique follows the protocol of CellsLife (Medellín, Colombia), a certified laboratory specialized exclusively in advanced biological therapies (Video 1). For processing SVF, approximately 40 mL of adipose tissue is harvested from the periumbilical region using a 13-gauge aspiration cannula connected to a syringe, under sterile conditions in a procedure room, with the patient under local anesthesia. This is then transported in a cold chain (2°C-8°C) to the CellsLife laboratory facilities, where processing begins under a Class II laminar flow biosafety cabinet (meeting International Organization for Standardization [ISO] 5 air cleanliness standards). SVF is obtained by washing to remove blood and tumescent fluid, as well as mechanical and enzymatic processing; it is composed of 1% to 10% mesenchymal cells, immune system cells, fibroblasts, endothelial cells, and others. Once SVF is obtained, it is packed in autologous platelet lysate. The processing time between the receipt of the adipose tissue sample and the delivery of SVF is 3 to 4 hours. After that, it can be sent to the treatment site for immediate application or application within the next 24 hours.

### Intraoperative Steps

**Fig 1:**
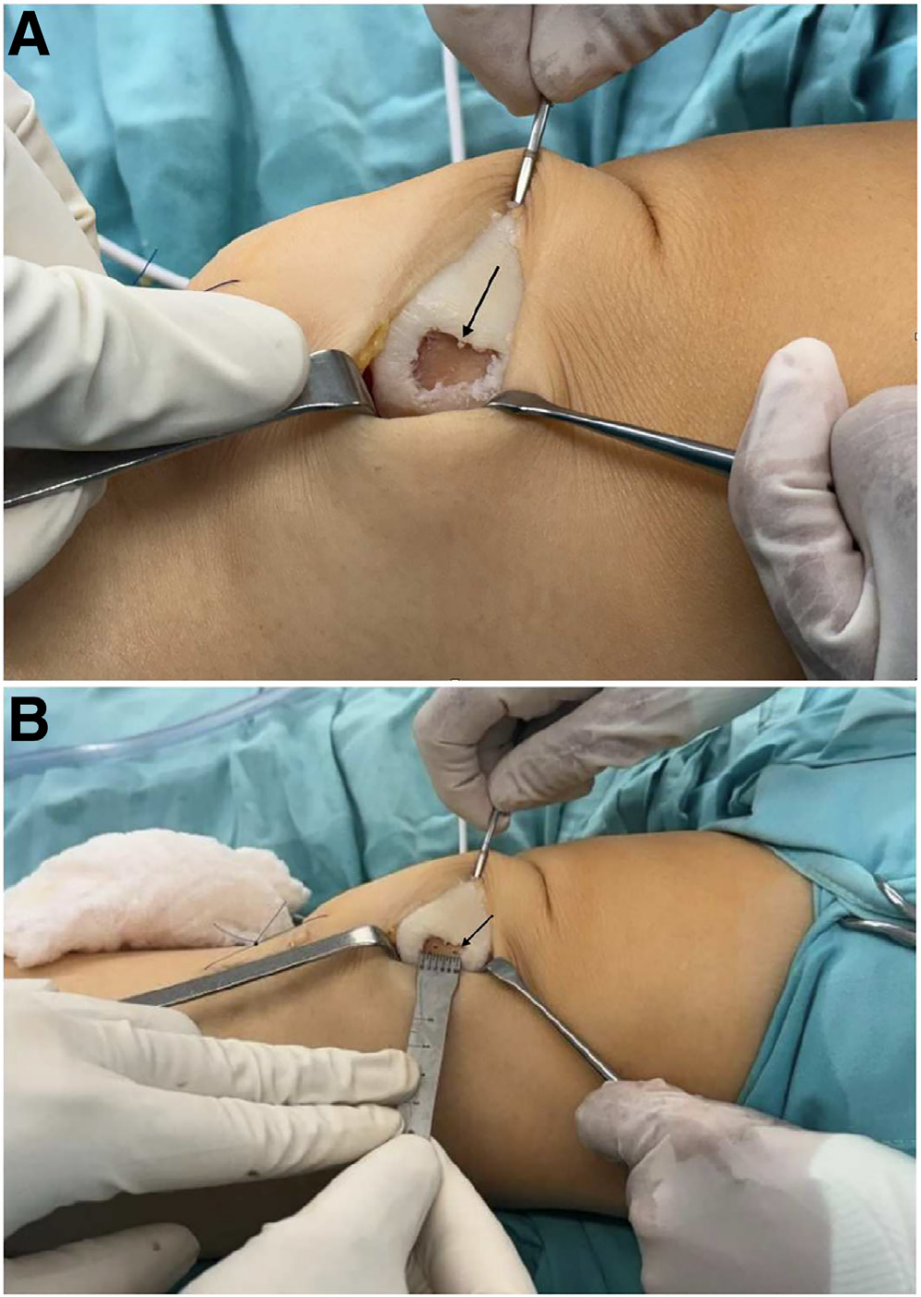
(A) Intraoperative image showing a mini-arthrotomy of the patient’s left knee. The patient is in the supine position, and the knee is extended. A focal cartilage lesion (arrow) on the patella is exposed. Resection of the unstable cartilage is performed using a rongeur and curette, leaving a focal lesion with well-defined edges, surrounded by healthy cartilage. (B) After the resection, precise measurement of the lesion is carried out using a millimeter ruler; subsequently, microfractures are created in the lesion bed (arrow) with a 1.2-mm pin to stimulate vascularization and cartilage regeneration.

The patient is placed in a supine position under regional anesthesia. A pneumatic tourniquet is applied to the proximal thigh, and the knee is positioned in extension to allow proper visualization of the patella. A mini-arthrotomy is performed to expose the articular surface. The focal cartilage lesion is identified, and unstable cartilage is removed using a rongeur and curette, creating a defect with well-defined, stable margins surrounded by healthy cartilage (Fig 1A).

Once the lesion is exposed, it is measured using a sterile millimeter ruler to select the appropriate scaffold size. Then, microfractures are created in the subchondral bone using a 1.2-mm pin, spaced approximately 3 to 4 mm apart. This step aims to stimulate vascular access and cell migration to enhance cartilage regeneration (Fig 1B).

**Fig 2:**
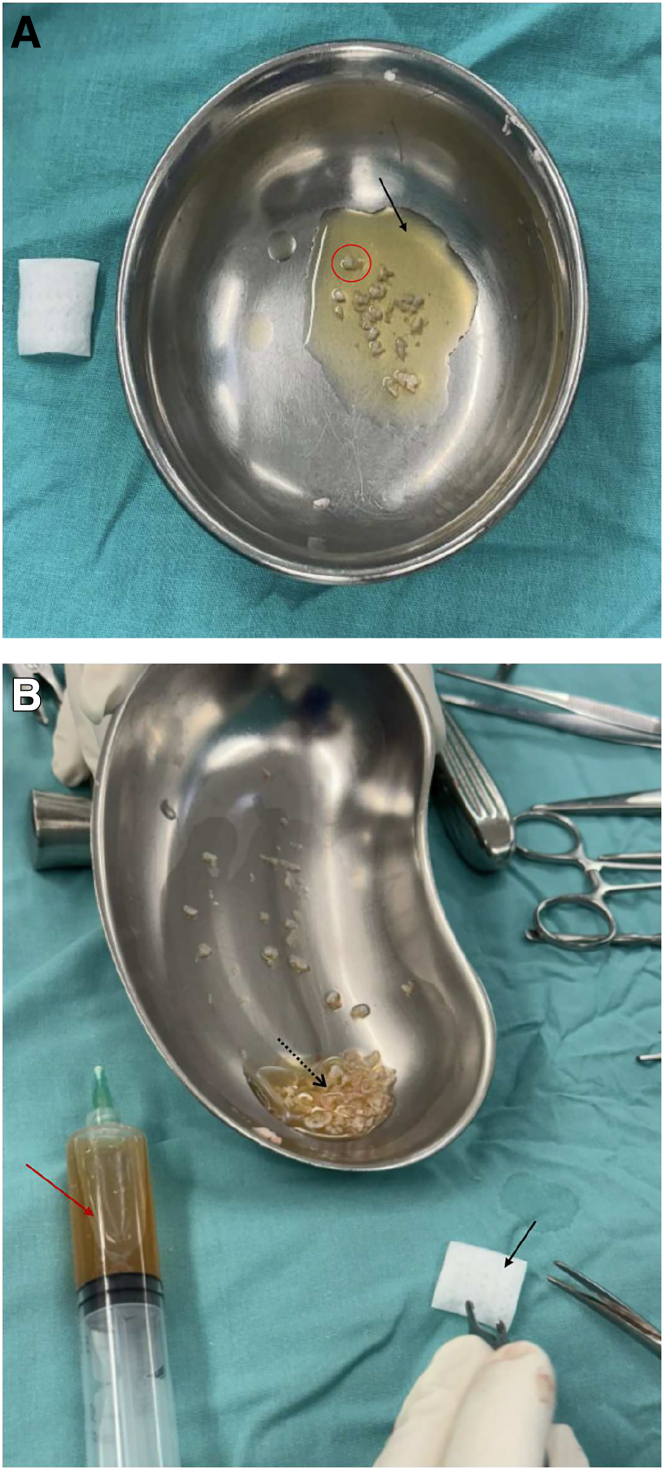
(A) Autologous cartilage obtained from the unstable area of the patellar lesion is selected, placed in a sterile tray, and manually minced into small fragments (circle) using a No. 11 scalpel. This cartilage was deemed to have the best macroscopic quality among the unstable tissue resected during lesion preparation. The particulates are then mixed with the stromal vascular fraction (SVF) (arrow), previously extracted from adipose tissue and prepared under sterile conditions, to generate a biologically active solution intended to enhance cartilage regeneration. (B) Final preparation of composite graft. The biological solution composed of autologous cartilage particulates and SVF is shown after mixing (dotted black arrow). On the sterile field, additional SVF is visible in a syringe (red arrow), and a hyaluronic acid mesh scaffold (Hyalofast) is present and ready for implantation (solid black arrow).

The resected cartilage is used to create autologous cartilage particulate by serial cutting with a No. 11 scalpel. Cartilage fragments with a macroscopically better appearance are selected and manually minced with care in a sterile tray. These particulates are then mixed with the SVF to form a biologically active composite aimed at creating a chondrogenic environment for the mesenchymal cells present in the SVF (Fig 2).

**Fig 3:**
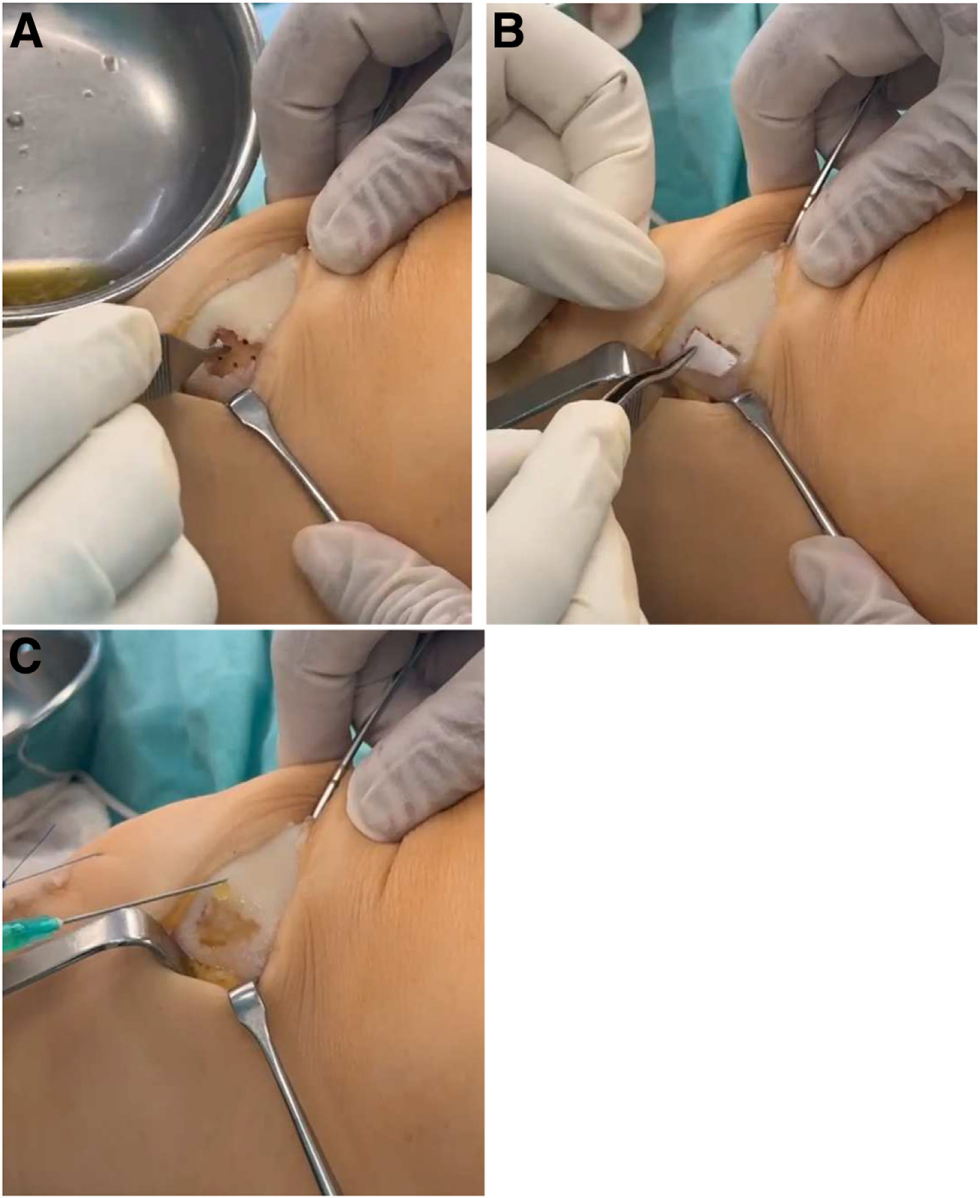
(A) Application of mixture. The mixture of autologous cartilage particulates and stromal vascular fraction (SVF) is applied directly to the focal cartilage lesion. This application is performed under direct visualization to ensure an even distribution of the mixture over the affected area. (B) Lesion coverage. A hyaluronic acid scaffold mesh is placed over the cartilage and SVF mixture. The mesh acts as a biological scaffold, providing structural support and facilitating cartilage regeneration at the lesion site. (C) Final application of remaining SVF. The remaining SVF is injected over the hyaluronic acid mesh and around the lesion, ensuring complete impregnation of the treated area and enhancing the regenerative effect.

The resulting mixture is applied directly to the prepared lesion bed under direct vision (Fig 3A). By use of a sterile instrument, the material is gently pressed to ensure an even and stable distribution over the microfractured surface. A 2 × 2-cm sheet of hyaluronic acid mesh scaffold (Hyalofast; Anika Therapeutics, Bedford, MA) is trimmed if needed and carefully positioned over the composite (Fig 3B). This scaffold serves to retain the biologic mixture in place and acts as a 3-dimensional matrix to support cell adhesion, organization, and integration into the lesion site. The remaining SVF is then injected over and around the scaffold to impregnate the treated area and further promote a regenerative microenvironment (Fig 3C).

**Fig 4:**
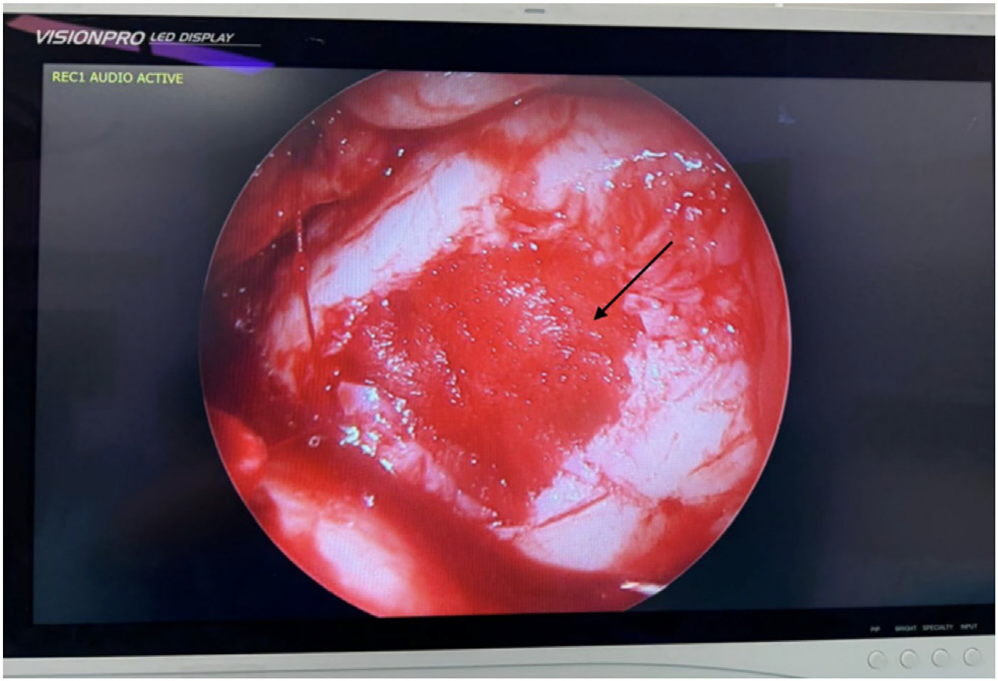
Dry arthroscopic view of scaffold (arrow). An arthroscopic view of the knee after the application of the hyaluronic acid mesh scaffold over the treated focal cartilage lesion. This image demonstrates the final appearance of the focal cartilage lesion after completing the surgical technique, showing proper adaptation and stability of the scaffold at the lesion site, confirming that the mixture of autologous cartilage particulates and stromal vascular fraction remains in place. The mini-arthrotomy site is used as the viewing portal.

A dry arthroscopic inspection is performed at the end of the procedure to confirm proper positioning and stability of the scaffold (Fig 4). After standard wound closure, the remaining volume of SVF in the syringe is injected intra-articularly to maximize local exposure of the joint environment to the bioactive cellular components. Table 1 presents a concise overview of technical recommendations, including key steps to ensure optimal graft integration, as well as potential intraoperative challenges.

**Table 1:** Key Technical Pearls and Pitfalls of Combined Use of SVF, Cartilage Particulates, and Hyaluronic Acid Scaffold for Focal Cartilage Repair

Pearls	Pitfalls
Use only cartilage that appears macroscopically healthy and stable.	Inadequate removal of unstable cartilage can compromise scaffold integration.
Space the microfractures evenly, approximately 3-4 mm apart, to ensure optimal subchondral bone stimulation without compromising structural integrity.	Excessive or uneven microfracturing may damage subchondral bone or reduce scaffold adherence.
Mix cartilage particulates and SVF beforehand to embed cells in a chondrogenic environment.	Omitting careful lesion bed preparation may impair graft adherence and compromise biological integration.
Trim and place the scaffold to match the lesion contour exactly.	Poor adaptation of the scaffold may allow graft migration or incomplete coverage.
Inject the remaining SVF intra-articularly after closure to enhance the biological milieu.	

SVF, stromal vascular fraction.

### Postoperative Care and Follow-Up

Postoperative management includes joint rest with the use of a rigid knee immobilizer in full extension for the first 2 weeks. During this period, patients are instructed to remove the immobilizer at least 5 times per day to perform gentle passive or active-assisted flexion exercises, gradually progressing up to 90°. If the treated lesion is located in a weight-bearing zone, partial or full weight-bearing is restricted for approximately 4 weeks. Physical therapy begins at 2 weeks postoperatively, focusing on progressive range of motion, quadriceps activation, and gait retraining under supervision.

The first clinical follow-up is scheduled at 2 weeks to assess wound healing and initiate the rehabilitation protocol. Subsequent evaluations are conducted monthly during the first 4 months or as clinically indicated. Radiographic imaging is obtained every 6 months, and magnetic resonance imaging is performed annually for up to 2 years to assess scaffold integrity, graft incorporation, and the degree of cartilage regeneration.

## Discussion

Preliminary results from this surgical technique, combining SVF with autologous cartilage particulate and hyaluronic acid mesh, show that it is a safe and feasible option for treating focal cartilage lesions. The addition of SVF provides a biologically active microenvironment that can enhance the viability and integration of particulate cartilage. A recent systematic review and meta-analysis of randomized controlled trials observed that for patients with knee osteoarthritis, intra-articular injection of SVF without adjunctive treatment showed notable clinical efficacy and safety in the short term.^5^

Considering that the intra-articular application of mesenchymal cells, which are an important part of the SVF, has been shown to improve function and quality of life in patients with patellofemoral osteoarthritis,^6^ the implementation of this technique could transform the management of cartilage injuries, especially in young and active patients seeking to preserve joint function. A detailed comparison of the advantages and disadvantages of this technique is summarized in Table 2, which may guide clinical decision making and patient selection.

**Table 2:** Advantages and Disadvantages of Single-Stage Cartilage Repair Technique Using SVF, Cartilage Particulates, and Scaffold

Advantages
Combines multiple biological elements to enhance cartilage repair
Single-stage technique with no need for cell expansion or culture
Scaffold provides structural support and maintains graft in place
Suitable for focal lesions in patients aiming to preserve native joint structures
Disadvantages
Requires access to specialized SVF processing facilities under GMP conditions
Cost of scaffold and SVF processing may be a barrier in some health care settings
Long-term data on repair tissue quality and durability are not yet available
Technique is currently limited to specialized centers with experience in biologic therapies

GMP, Good Manufacturing Practices; SVF, stromal vascular fraction.

Recent studies have further validated the clinical efficacy and safety of SVF for cartilage lesions. Kim et al.^7^ showed superior outcomes in pain relief and cartilage regeneration in patients receiving arthroscopic treatment combined with SVF implantation compared with conventional arthroscopic treatment alone, with significant improvements observed through magnetic resonance imaging evaluations at 12 months. Similarly, a recent meta-analysis by Han et al.^8^ confirmed that intra-articular SVF injections provide consistent and clinically meaningful improvements in both pain and functional scores when compared with hyaluronic acid and saline solution injections, although the advantages over corticosteroids were less clear, especially in the short term. These findings reinforce the potential benefits of SVF in enhancing cartilage repair outcomes, emphasizing its role as a viable therapeutic approach that leverages the synergistic interaction between regenerative cells and extracellular matrix fragments. The described technique offers a unique combination of autologous biologic elements—cartilage particulates, mesenchymal cells, and hyaluronic acid—that together create a chondrogenic microenvironment that supports cellular adhesion, migration, and differentiation.

One of the drawbacks associated with the use of SVF is the increased cost of the procedure, primarily because of the need for specialized laboratory processing. However, by offering significant regenerative effects, it has the potential to slow or even halt disease progression.^9^ Additionally, the minimally invasive harvesting of adipose tissue and the low incidence of adverse effects, as shown in recent trials, support its safety profile.^2^ No serious complications have been reported with SVF use, aside from transient local symptoms at the liposuction site, such as pain or swelling. In this context, the benefits of biologically enhanced repair may outweigh the upfront costs, particularly in selected patient populations in whom joint preservation is a primary goal. By leveraging these regenerative properties, more invasive and costly procedures could be avoided or delayed in the future.

## Disclosures

The authors (G.A., V.C., F.S., S.L., A.Z., C.C., A.A.) declare no financial or personal relationships that could be considered as potential competing interests.

## Supplementary Data

Video 1 Surgical technique demonstrating the treatment of a focal cartilage lesion using autologous cartilage particulate, stromal vascular fraction (SVF), and a hyaluronic acid-based membrane. The patient’s right knee is positioned in extension with the patient in the supine position. The lesion is identified and debrided through a lateral mini-arthrotomy to create stable margins. Microfractures are performed using a 1.2 mm awl. Autologous cartilage particulates are prepared and mixed with SVF, then applied to the defect and covered with a hyaluronic acid-based membrane. The remaining SVF is injected over and around the graft. A dry arthroscopic evaluation is performed through the mini-arthrotomy to confirm the stability of the construct.
